# Retraction: Acute Toxicity and Gastroprotection Studies with a Newly Synthesized Steroid

**DOI:** 10.1371/journal.pone.0294010

**Published:** 2023-11-10

**Authors:** The PLOS ONE Editors

Following the publication of this article [1], concerns were raised regarding reuse of results presented in Figs 2 and 6 in subsequent publications. Specifically,

- Fig 2A of this study [1] appears similar to Fig 2A of [2, retracted in 3] despite being used to represent different experimental conditions.
- Fig 6A of this study [1] appears similar to Fig 7B of [4, retracted in 5] despite being used to represent different experimental conditions.
- Fig 6D of this study [1] appears similar to the following results, despite being used to represent different experimental conditions:
  ○ Fig 8a of [6, retracted in 7]
  ○ Fig 6 panel G1 of [8, retracted in 9]
  ○ Fig 10A of [10]
  ○ Fig 5a of [11]

The similar (or reused) images were used to represent controls in each of the articles, but different methodological details were reported for the indicated experiments in the articles’ Materials and Methods sections. Given the nature and extent of the issues, the *PLOS ONE* Editors are concerned about the reliability of data management and/or reporting for this study [1].

Following editorial communication regarding these concerns, one of the co-authors requested retraction of the article. The original data underlying this study were not provided for editorial review.

In light of the concerns affecting multiple figure panels that question the integrity of these data, the *PLOS ONE* Editors retract this article.

SG, PH, NAM, and MAA agreed with the retraction. MH responded but expressed neither agreement nor disagreement with the editorial decision. KAK, AHAH, and HMA either did not respond directly or could not be reached. SG and NAM apologize for the issues with the published article. MAA stands by the article’s findings.
